# Anxiety is associated with appetitive traits in university students during the COVID-19 pandemic

**DOI:** 10.1186/s12937-021-00701-9

**Published:** 2021-05-13

**Authors:** Kathryn E. Coakley, Huyen Le, Spirit Rae Silva, Aspen Wilks

**Affiliations:** College of Education & Human Sciences, University of New Mexico, Albuquerque, NM 87131 USA

**Keywords:** Anxiety, Mental health, Appetitive traits, Hunger, Emotional eating, Undergraduate students, Graduate students, COVID-19

## Abstract

**Background:**

COVID-19 has impacted mental health globally, however, associations between anxiety and appetitive traits during the pandemic are unreported. This study evaluated anxiety symptom severity and associations with appetitive traits in students at a large public University in the U.S. during the pandemic.

**Methods:**

Current undergraduate and graduate/professional students completed a cross-sectional survey in fall 2020. Demographic information, anxiety symptoms in the past 2 weeks assessed by the Generalized Anxiety Disorder Scale (GAD-7), and appetitive traits assessed by the Adult Eating Behavior Questionnaire (AEBQ) were evaluated. Mean scores for eight AEBQ scales (four food approach and four food avoidance traits) were calculated. Differences in mean scores were examined between participants with moderate to severe anxiety symptoms (GAD-7 score ≥ 10) and those with mild to no anxiety symptoms (GAD-7 score < 10) via independent samples t-tests and effect sizes. Associations between GAD-7 score and individual appetitive traits were also examined, adjusting for age and gender.

**Results:**

Of the 1243 students who completed the survey (57% undergraduates; mean age = 26.5 years), 51.9% reported moderate to severe anxiety symptoms. Groups experiencing the highest degree of moderate to severe anxiety symptoms included transgender, gender fluid, and other-gendered participants (73.6%); the youngest age group [18–20 years (62%)]; undergraduate students (60.7%); and Hispanic/Latinx participants (57.7%). Participants with moderate to severe anxiety symptoms had higher scores for most food approach and avoidance traits but lower scores for enjoyment of food than those with mild to no anxiety symptoms. Effect sizes were largest for hunger and emotional over-eating (Cohen’s *d* = 0.31 and 0.30, respectively). Adjusting for age and gender, GAD-7 score was significantly and positively associated with hunger, emotional over-eating, food and satiety responsiveness, and food fussiness and negatively associated with enjoyment of food.

**Conclusions:**

Over half of students at a U.S. University reported moderate to severe anxiety symptoms during COVID-19. More severe anxiety symptoms were associated with increased hunger, emotional over-eating, and food and satiety responsiveness and decreased enjoyment of food. Universities must consider strategies to address anxiety, particularly in younger students; transgender, gender fluid, and students of other genders; and across race/ethnicities keeping in mind associations with appetitive traits.

**Supplementary Information:**

The online version contains supplementary material available at 10.1186/s12937-021-00701-9.

## Background

The novel coronavirus 2019 (COVID-19) pandemic has impacted mental health around the world [1]. Fear of contracting the virus combined with the implementation of public health measures including stay-at-home orders, lockdowns, quarantining and self-isolation have caused significant stress and anxiety [2, 3]. By June 2020, the Centers for Disease Control and Prevention (CDC) indicated over 40% of the general population in the U.S. reported one or more adverse mental or behavioral health symptom [4]. Prior to the COVID-19 pandemic, anxiety disorders were the most common mental illness in the U.S., affecting 18.1% of the population 18 years and older [5]. In June 2020, more than one in four U.S. adults reported symptoms indicating an anxiety disorder [4]. Rising rates of anxiety are not, however, unique to the United States. A systematic review examining the impact of COVID-19 on mental health found symptoms of anxiety in 6.33 to 50.9% of the general population worldwide [6]. Country-specific estimates vary widely. In Italy, over 70% of adults reported anxious feelings at the start of the pandemic [7]. In Spain, 12.3% reported symptoms of anxiety shortly after lockdown measures while in Greece, 13.2% reported anxiety symptoms after lockdown measures [8].

Young adults have experienced high degrees of anxiety during the COVID-19 pandemic. Almost half of young adults 18–24 years of age in the U.S. had symptoms of anxiety by June 2020 compared to 25.5% of the general population [4]. In Portugal, the highest scores for anxiety were found in the youngest age group (18–34 years) [9]. In Canada, 65.5% of young adults displayed symptoms consistent with generalized anxiety disorder (GAD), much higher than 23.3% of adults over 60 years of age [10]. Over 40% of youth across six middle eastern countries had anxiety in April 2020 [11]. Young adults attending college or university experienced the added stress of campuses closing and, at many institutions, the transition to remote and online learning [12, 13]. University students reported difficultly coping with pandemic-related stress adequately [14, 15]. In the U.S., 71% of college students indicated their stress and anxiety increased due to the pandemic [14]. Worldwide, estimates of the burden of anxiety in university students during the early months of the COVID-19 pandemic ranged from 25% in China [16] to 27.5% in France [17] to 38.5% at a single U.S. University [15] to 42.9% in Bangladesh [18].

Short and long-term impacts of pandemic-induced stress and anxiety are also emerging. Stress and anxiety are known triggers for undereating, overeating, and other maladaptive eating attitudes and behaviors [19, 20]. In college students, anxiety and depression are associated with changes in food choices and total caloric intake [21]. In undergraduate students, COVID-19-related public health measures including quarantining have been associated with concerns about body weight and shape and eating habits [22]. Adults with and without eating disorders have reported increased restrictive behaviors, fears regarding finding food, and increased urges to binge and actual binge eating episodes during the pandemic [23, 24]. Early evidence suggests the COVID-19 pandemic has also impacted appetitive traits [25, 26]. Appetitive traits are predispositions toward food that may interact with environmental factors to influence dietary behaviors and consequences such as weight gain or loss [26]. Pandemic-induced anxiety in particular is tied to maladaptive eating behaviors [8]. Italian adults who felt anxious reported consuming comfort food and increased food intake at the start of the pandemic [7]. In summary, the COVID-19 pandemic has created an environment of stress, worry, and anxiety, likely impacting appetitive traits and dietary behaviors.

Despite the rise in mental distress and anxiety worldwide, there no evidence on relationships between anxiety and appetitive traits in university students during the COVID-19 pandemic. This study aimed to examine anxiety symptoms in university students amidst the COVID-19 pandemic and associations with food approach and food avoidance appetitive traits.

## Methods

A cross-sectional survey-based study was conducted during the COVID-19 pandemic at a large, public University in the southwest region of the U.S. The authors followed the STROBE-nut Statement to report results [27].

### Participants and recruitment

Inclusion criteria for this study were: currently enrolled undergraduate or graduate/professional student at the University and at least 18 years of age. Participants were recruited for 4 weeks from September to October 2020 to examine anxiety and appetitive traits mid-pandemic. A recruitment email was distributed to all undergraduate and graduate students currently enrolled in each of the University’s Colleges; one College declined to distribute the email. The recruitment email was also sent via the University’s Graduate and Professional Student listserv. Participants provided consent by starting the survey and no consent signature was required. At the end of the survey, students could choose to provide their email address to enter a drawing for a $20 gift card incentive. Five email addresses were randomly selected to receive $20 gift cards after data were collected. The study was approved by the University’s Office of the Institutional Review Board.

### Data collection

Data were collected via Opinio, an online survey tool approved for research. The survey included two screening questions assessing the potential participant’s age and confirming current enrollment as a full-time or part-time undergraduate or graduate/professional student at the University. The survey then included four demographic questions (gender, age, race/ethnicity, and academic status); the seven-item Generalized Anxiety Disorder Scale (GAD-7), a validated measure of symptoms of anxiety in the past 2 weeks (Cronbach’s α = 0.89) [28, 29]; and the 35-item Adult Eating Behavior Questionnaire (AEBQ), a validated measure of eight appetitive traits (Cronbach’s α range = 0.75–0.90) [30, 31]. No other demographic or health-related information was collected from participants. The full survey is available in Additional file 1.

The GAD-7 was designed to identify probable cases of GAD and to assess symptom severity through seven statements (e.g. feeling nervous, anxious, or on edge; trouble relaxing), each rated from 0 = not at all to 3 = nearly every day [28]. Scores range from 0 to 21 and higher scores indicate more severe anxiety symptoms. For descriptive purposes, anxiety symptom severity was categorized based on standard scoring criteria as no symptoms of anxiety (GAD-7 score < 5), mild anxiety symptoms (GAD-7 score 5–9), moderate anxiety symptoms (GAD-7 score 10–14), or severe anxiety symptoms (GAD-7 score ≥ 15) [28]. GAD-7 scores were also dichotomized to mild to no anxiety symptoms (GAD-7 score < 10) or moderate to severe anxiety symptoms (GAD-7 score > 10) for analyses [28].

The AEBQ is a validated measure of appetitive traits and includes eight scales divided into four food approach traits (hunger, food responsiveness, emotional over-eating, and enjoyment of food) and four food avoidance traits (satiety responsiveness, emotional under-eating, food fussiness, and slowness in eating) [31]. Each of the 35 AEBQ statements are assessed via Likert Scale (1 = strongly disagree to 5 = strongly agree), then items are combined into the eight scales representing individual appetitive traits. The AEBQ hunger scale represents physical hunger and includes five statements (e.g. I often notice my stomach rumbling) [31]. Food responsiveness is the tendency to eat (or eat more) in response to food cues including the sight and smell of food and is assessed through five statements (e.g. I am always thinking about food) [32]. Emotional over-eating is defined as eating more in response to negative emotions, assessed through five statements (e.g. I eat more when I’m anxious) [30]. Enjoyment of food represents enjoyment related to eating, assessed through three statements (e.g. I enjoy eating) [33]. Satiety responsiveness is defined as the capacity to adjust eating and food intake in response to feelings of satiety and fullness, assessed through four statements (e.g. I get full easily) [32]. Emotional under-eating represents eating less in response to negative emotions, assessed through five statements (e.g. I eat less when I’m annoyed) [31]. Food fussiness is defined as selectivity about the range of foods that are acceptable, assessed through five statements (e.g. I refuse new foods at first) [33]. Finally, slowness in eating represents speed of eating including dawdling or taking a long time to finish a meal, assessed through four statements (e.g. I eat slowly) [33].

### Data analysis

A power analysis was not conducted due to the exploratory nature of the study. All participants who completed the GAD-7 were included in data analyses. Since all survey questions were optional, some demographic data were missing and are reported as such. All data analyses were performed using Statistical Analysis Software (SAS; version 9.4; SAS Institute, Cary, NC, USA) and all *p*-values calculated are included in results.

Demographic data were presented descriptively. Gender was collapsed into three categories: female; male; or non-cisgender which included transgender, gender fluid, or any other gender identity. The AEBQ was scored according to standard guidelines by reverse scoring four of the 35 statements and then calculating the average of Likert scale responses (scale 1–5) for questions included in each of the eight scales [31]. Differences in the percent of respondents reporting no symptoms of anxiety, mild symptoms of anxiety, moderate symptoms of anxiety, and severe symptoms of anxiety were examined by demographic subgroups including age category, gender, race/ethnicity, and student status (undergraduate or graduate/professional) using Chi-square tests. Differences in mean AEBQ scores were compared between participants with mild to no symptoms of anxiety and those with moderate to severe symptoms of anxiety using independent samples t-tests. Cohen’s *d* was also calculated to examine effect sizes for the difference in mean appetitive trait scores between the two groups. Unadjusted associations between individual AEBQ scale scores and GAD-7 score were examined by calculating r-squared values (r^2^) via linear regression. Multivariate linear regression was then utilized to calculate beta coefficients (β) examining associations between individual AEBQ scale scores and GAD-7 score adjusting for age (continuous) and gender (categorical). Finally, a full model including all AEBQ scales as predictors was considered to examine the percent of variation (R^2^) in GAD-7 score explained by the eight appetitive traits.

## Results

Eight of the nine Colleges at the University and the Graduate and Professional Student Association sent the recruitment email to all currently enrolled undergraduate and graduate/professional students in September 2020. The number of students who received the recruitment email was not documented; however, 1367 students started the survey. Of those, 17 (1.2%) were excluded based on responses to the two screening questions and an additional 107 (7.8%) were excluded for not completing the GAD-7. Thus, 1243 (90.9%) participants were included in the final analysis.

Demographic characteristics of the study sample are presented in Table 1. The mean age of participants was 26.5 years (SD = 9.2; range 18–82). The majority were female (*n* = 909; 73.1%), white (*n* = 732; 59.2%) or Hispanic/Latinx (*n* = 239; 19.3%), and undergraduate students (*n* = 702; 57%). The study sample differed from the general population of the University in fall 2020, with an overrepresentation of females (73.1% versus 57.4%) and white students (59.2% versus 33.3%) and an underrepresentation of undergraduate students (57% versus 70.9%). The study sample did, however, accurately represent the University’s composition of American Indian or Alaska Native students and Black or African American students. Mean age in this study (26.5 years) was comparable to the University’s overall average of 25.7 years, reflecting the large population of non-traditional students.

**Table 1:** Demographic characteristics of study participants (*n* = 1243)

Demographics^a^	n	%
Age (years)		
18–20	353	29.5
21–24	326	27.2
25–30	205	17.1
> 30	314	26.2
Gender		
Female	909	73.1
Male	281	22.6
Non-cisgender (transgender, gender fluid, other)	53	4.3
Race/Ethnicity		
American Indian or Alaska Native	60	4.9
Asian	99	8.0
Black or African American	27	2.2
Hispanic/Latinx	239	19.3
White	732	59.2
More than one race/ethnicity or other	79	6.4
Student Status		
Undergraduate	702	57.0
Graduate/Professional	530	43.0
Anxiety Symptom Severity (GAD-7 score)		
No symptoms of anxiety (< 5)	238	19.2
Mild symptoms of anxiety (5–9)	360	29.0
Moderate symptoms of anxiety (10–14)	339	27.3
Severe symptoms of anxiety (> 15)	306	24.6

^a^Age missing for 45 participants; race/ethnicity missing for 7 participants; student status missing for 11 participants

In the full sample, the mean GAD-7 score was 10.1 (SD = 5.6). Over half of participants (*n* = 645; 51.9%) reported moderate to severe symptoms of anxiety in the past 2 weeks. In the full sample, 29% had mild symptoms of anxiety, 27.3% had moderate symptoms of anxiety, and 24.6% had severe symptoms of anxiety. Nearly 90% of participants indicated symptoms of anxiety made it somewhat difficult (54.9%), very difficult (22.2%), or extremely difficult (10.9%) to do work, take care of things at home, or get along with other people. Anxiety symptom severity differed significantly by age category, gender, and level of education but not by race/ethnicity (Table 2). The youngest age category (18–20 years of age) had the highest degree of moderate symptoms (33.7%) and severe symptoms of anxiety (28.3%) compared to older age groups. The oldest age category (> 30 years of age) reported the lowest degree of moderate symptoms (20.4%) and severe symptoms of anxiety (19.4%). Transgender, gender fluid, and other-gendered participants had the highest degree of moderate symptoms (34%) and severe symptoms of anxiety (39.6%) compared to females and males. Male participants had the lowest degree of moderate and severe symptoms of anxiety.

**Table 2:** Anxiety symptom severity in university students by demographic characteristics

	No anxiety symptoms (GAD-7 score < 5)		Mild anxiety symptoms (GAD-7 score 5–9)		Moderate anxiety symptoms (GAD-7 score 10–14)		Severe anxiety symptoms (GAD-7 score > 15)		
	n	%	n	%	n	%	n	%	*p*^a^
Age (years)									< 0.01
18–20	52	14.7	82	23.2	119	33.7	100	28.3	
21–24	50	15.3	102	31.3	91	27.9	83	25.5	
25–30	38	18.5	62	30.2	56	27.3	49	23.9	
> 30	88	28.0	101	32.2	64	20.4	61	19.4	
Gender									< 0.01
Female	145	16.0	269	29.6	260	28.6	235	25.9	
Male	90	32.0	80	28.5	61	21.7	50	17.8	
Transgender, gender fluid, other	3	5.7	11	20.8	18	34.0	21	39.6	
Race/Ethnicity									0.13
American Indian or Alaska Native	8	13.3	19	31.7	17	28.3	16	26.7	
Asian	30	30.3	32	32.3	20	20.2	17	17.2	
Black or African American	6	22.2	6	22.2	7	25.9	8	29.6	
Hispanic/Latinx	31	13.0	70	29.3	69	28.9	69	28.9	
White	149	20.4	207	28.3	202	27.6	174	23.8	
> 1 Race/Other	13	16.5	22	27.9	24	30.4	20	25.3	
Student Status									< 0.01
Undergraduate	98	14.0	178	25.4	223	31.8	203	28.9	
Graduate/ Professional	140	26.4	178	33.6	112	21.1	100	18.9	

^a^Chi-square test for difference in anxiety symptom severity (no anxiety, mild anxiety, moderate anxiety, severe anxiety) within demographic groups

In unadjusted analyses, undergraduate students reported a higher degree of moderate to severe anxiety symptoms (60.7%) compared to graduate students (40.0%). While anxiety severity was not statistically significantly different by race/ethnicity (*p* = 0.13), Asian participants reported the lowest degree of moderate to severe anxiety symptoms (37.4%) compared to all other race/ethnicity groups (> 50%). Hispanic/Latinx participants had the highest degree of moderate to severe anxiety symptoms (57.7%), followed by students reporting other or more than one race/ethnicity (55.7%). Severe anxiety symptoms were reported to the highest degree in Black or African American students (29.6%), followed by Hispanic/Latinx students (28.9%).

Table 3 shows mean AEBQ scores in the full sample and by anxiety symptom severity (mild to no anxiety symptoms compared to moderate to severe anxiety symptoms). Mean scores for food approach scales (hunger, food responsiveness, emotional over-eating) were significantly higher in those with moderate to severe symptoms of anxiety, except enjoyment of food which was significantly lower. Mean scores for three of the four food avoidance scales (satiety responsiveness, emotional under-eating, and food fussiness) were significantly higher in those with moderate to severe symptoms of anxiety compared to those with mild to no symptoms of anxiety. According to Cohen’s guidelines, effect sizes were small to medium for food approach traits and small for food avoidance traits [34]. The largest effect sizes were found for hunger (0.31), emotional over-eating (0.30), and enjoyment of food (0.25) and food responsiveness (0.25). The smallest effect sizes were found for slowness in eating (0.08), emotional under-eating (0.16), and satiety responsiveness (0.17).

**Table 3:** Appetitive traits in the full sample and by anxiety symptom severity

	All Participants (*n* = 1243)		Mild to no anxiety symptoms (*n* = 598)		Moderate to severe anxiety symptoms^a^ (*n* = 645)			Effect Size
AEBQ Scales	Mean	SD	Mean	SD	Mean	SD	*p*^b^	Cohen’s *d*
Food approach traits								
Hunger	3.10	0.75	2.98	0.74	3.21	0.75	< 0.01	0.31
Food responsiveness	3.18	0.75	3.08	0.73	3.27	0.75	< 0.01	0.25
Emotional over-eating	2.85	1.01	2.69	0.93	2.99	1.05	< 0.01	0.30
Enjoyment of food	4.14	0.80	4.24	0.71	4.04	0.86	< 0.01	0.25
Food avoidance traits								
Satiety responsiveness	2.74	0.82	2.66	0.77	2.81	0.86	< 0.01	0.17
Emotional under-eating	3.11	0.96	3.03	0.89	3.18	1.02	< 0.01	0.16
Food fussiness	2.03	0.84	1.95	0.79	2.11	0.88	< 0.01	0.19
Slowness in eating	2.71	1.01	2.66	0.94	2.75	1.06	0.23	0.08

^a^Moderate to severe symptoms of anxiety defined as GAD-7 score > 10; ^b^Independent samples t-tests with equal variance comparing AEBQ scores by anxiety symptom severity category

In unadjusted analyses, seven of the eight AEBQ scales were significantly associated with GAD-7 score (Table 4). Adjusting for age and gender, six of the eight scales remained significantly associated: hunger, food responsiveness, emotional over-eating, satiety responsiveness, and food fussiness were significantly positively associated with GAD-7 score while enjoyment of food was significantly negatively associated with GAD-7 score. Hunger (β = 1.69), emotional over-eating (β = 1.02), and enjoyment of food (β = − 0.84) and satiety responsiveness (β = 0.84) had the strongest associations with GAD-7 score. A multivariate regression model with GAD-7 score as the outcome and all eight appetitive traits as predictors revealed an R^2^ of 0.155, suggesting the eight AEBQ scales explained approximately 15.5% of the variability in GAD-7 score. In the full model, all scales were significantly associated with GAD-7 score except food fussiness and slowness in eating.

**Table 4:** Associations between appetitive traits and GAD-7 score

AEBQ Scales	Unadjusted^a^		Adjusted^b^		Full model^c^ (R^2^ = 0.155)	
	β	*p*	β	*p*	β	*p*
Food approach traits						
Hunger	1.79	< 0.01	1.69	< 0.01	1.36	< 0.01
Food responsiveness	0.92	< 0.01	0.81	< 0.01	0.58	0.03
Emotional over-eating	1.00	< 0.01	1.02	< 0.01	1.63	< 0.01
Enjoyment of food	−0.87	< 0.01	−0.84	< 0.01	−1.31	< 0.01
Food avoidance traits						
Satiety responsiveness	0.92	< 0.01	0.84	< 0.01	0.54	0.01
Emotional under-eating	0.40	0.02	0.32	0.06	1.07	< 0.01
Food fussiness	0.66	< 0.01	0.55	< 0.01	0.20	0.28
Slowness in eating	0.22	0.16	0.17	0.28	0.01	0.95

^a^Associations between individual traits and GAD-7 score; ^b^Associations between individual traits and GAD-7 score, adjusted for age and gender; ^c^Full model with all appetitive traits as predictors of GAD-7 score

## Discussion

Over half of a large sample of undergraduate, graduate, and professional students at a University in the southwest U.S. reported moderate to severe symptoms of anxiety in fall 2020 amidst the COVID-19 pandemic. The percent of students reporting moderate to severe symptoms of anxiety in this study was higher than estimates from spring and summer 2020 which ranged from 25 to 43% in college students around the world suggesting the pandemic may have lasting effects on mental health [16, 18]. Moderate to severe anxiety symptoms were most worrisome in the youngest group of participants 18–20 years of age; undergraduate students compared to graduate and professional students; and transgender, gender fluid, and other-gendered participants. Other studies have also tied younger age to more severe mental health symptoms during the COVID-19 pandemic [4, 9, 10, 35]; it is no surprise that more undergraduate students experienced moderate to severe anxiety symptoms compared to graduate and professional students. Findings from this study also add to literature suggesting transgender and people whose biological sex is different from their current gender identity report elevated symptoms of anxiety, depression, and substance use disorders [36, 37]. During the pandemic, anxiety and worry may be additionally elevated in these groups due to hesitations seeking healthcare treatment for fear of mistreatment or discrimination and potentially increased risk of illness and mortality [38]. As the COVID-19 pandemic lingers, a global mental health crisis is hypothesized [39] and, based on mounting evidence, may disproportionately affect young adults and people who identify as transgender, gender fluid, non-binary, or genders other than male or female [4].

This study suggests anxiety symptom severity is associated with appetitive traits in university students. Participants with moderate to severe symptoms of anxiety had higher scores for food approach and food avoidance traits, except enjoyment of eating which was significantly lower than those with mild to no anxiety symptoms. Effect size estimates suggest the magnitude of difference between the two groups was greater for food approach traits (hunger, emotional over-eating, food responsiveness, and enjoyment of food), though still small to medium. Participants in this study with moderate to severe symptoms of anxiety had higher mean scores for emotional over-eating, emotional under-eating, and hunger compared to a study examining the AEBQ in the general population in Australia and the United Kingdom prior to the COVID-19 pandemic [30]. Another study of Greek and Spanish adults conducted in April 2020 during the COVID-19 pandemic also found high scores for maladaptive eating attitudes and behaviors including restrained eating, external eating, and emotional eating [8].

Effect size estimates and adjusted regression coefficients suggest food approach traits had a stronger association with anxiety symptom severity than food avoidance traits. For example, a one-unit increase in AEBQ hunger scale score corresponded with a 1.69-point increase in GAD-7 score. Food avoidance traits were associated with GAD-7 score, but to a lesser degree. A one-unit increase in AEBQ satiety responsiveness scale score, for example, corresponded to a 0.84-point increase in GAD-7 score. Previously published evidence also suggests emotional over-eating and emotional under-eating are responses to anxiety [40]. Effects of anxiety on maladaptive appetitive traits may be even more pronounced due to the additional stress and worry of the COVID-19 pandemic [2, 9, 23, 41]. Moreover, there may be a bi-directional relationship between anxiety and appetitive traits. While more severe anxiety symptoms could lead to changes in appetitive traits, particularly food approach traits, it could very well be that maladaptive appetitive traits could increase anxiety symptom severity [42]. Additional longitudinal research must address the directionality of associations between anxiety symptoms and appetitive traits.

In this study, GAD-7 scores were associated with most AEBQ scale scores but individual effect sizes were small to medium. Together, the eight AEBQ scales did, however, explain nearly 16% of the variability in GAD-7 score. Particularly concerning is the positive association between GAD-7 score and hunger indicating increasing anxiety severity is associated with increases in physical hunger. This association was the strongest found among the eight appetitive traits (Cohen’s *d* = 0.31). Symptoms of anxiety reported in this study could stem from a number of environmental factors caused by the COVID-19 pandemic ranging from the closing of the University’s campus, to job loss or furlough, to food insecurity [43]. Food insecurity by definition includes uncertainty and worry, similar to symptoms of anxiety included in the GAD-7 [28, 44]. Very low food security, the most severe category of food insecurity, is characterized by disruptions in food intake including skipping or cutting meals which could result in hunger [44]. The intricate relationships between anxiety, food insecurity, and physical hunger must continue to be explored during and after the COVID-19 pandemic.

Increasing anxiety severity was also associated with increases in emotional over-eating and food and satiety responsiveness scores. Food responsiveness is the tendency to eat more in response to food cues, potentially in excess of requirements. A similar study conducted during the COVID-19 pandemic also suggests GAD-7 scores are significantly correlated with Dutch Eating Behavior Questionnaire (DEBQ) scales including restrained eating, emotional eating, and external eating [8]. It is important to note this study assessed appetitive traits, not actual eating behaviors, and additional research is necessary to examine associations between appetitive traits and eating behaviors, especially since the pandemic is hypothesized to increase the risk of eating disorders and the severity of symptoms [45].

### Limitations

Limitations must be considered when interpreting results of this study. All data were self-reported and certain demographic groups (females, white participants, and undergraduate students) were overrepresented. Generalizability may be limited to university students; however, the sample included a diverse group of participants. Data were also missing for demographic questions including 45 missing responses for age. An exact response rate could not be calculated since the exact number of students receiving the recruitment email was not determined. Moreover, measures of disordered eating behaviors or eating disorder diagnoses were not assessed. COVID-19 related variables including self-isolating, quarantining, or testing positive for SARS-CoV-2 were also not measured. Finally, given the cross-sectional design of the study, it is impossible to determine if anxiety symptoms may have led to maladaptive appetitive traits or if appetitive traits increased anxiety symptom severity during the COVID-19 pandemic. The directionality of the relationship between anxiety and appetitive traits, particularly hunger, emotional over-eating, and food and satiety responsiveness must be explored in future longitudinal studies.

## Conclusions

Results of this study add to evidence suggesting a mental health crisis during the COVID-19 pandemic, particularly in younger university students and those who identify as a gender other than male or female. University students with symptoms of moderate to severe anxiety reported higher degrees of maladaptive appetitive traits including hunger, food and satiety responsiveness, and emotional over-eating, and less enjoyment of food than those with mild to no symptoms of anxiety. Anxiety severity was also associated with increases in hunger, emotional over-eating, and food and satiety responsiveness, and decreases in enjoyment of food. As professionals consider interventions to prevent and treat COVID-19 related mental health concerns in university students, disparities in anxiety symptom severity and associations with appetitive traits must be considered.

## Supplementary Information

**Additional file 1.** “Survey Instrument” includes the full survey instrument used in this study.

## Data Availability

The dataset analyzed during the current study is available from the corresponding author on reasonable request.

## Abbreviations

COVID-19: Coronavirus disease 2019
CDC: Centers for Disease Control and Prevention
GAD: Generalized anxiety disorder
STROBE-nut: Strengthening the Reporting of Observational Studies in Epidemiology – Nutritional Epidemiology
GAD-7: Generalized Anxiety Disorder Scale
AEBQ: Adult Eating Behavior Questionnaire
SAS: Statistical Analysis Software
DEBQ: Dutch Eating Behavior Questionnaire
