# Supplementary material for: Anxiety is associated with appetitive traits in university students during the COVID-19 pandemic
Source: Nutr J. 2021 May 13;20:45. doi: 10.1186/s12937-021-00701-9 (PMC8118620; doi:10.1186/s12937-021-00701-9)
Supplement: Supplementary file 1 — Additional file 1. “Survey Instrument” includes the full survey instrument used in this study. [file 12937_2021_701_MOESM1_ESM.pdf]

Survey Instrument

1. Currently, how do you describe yourself? You may select more than one.
   ____ Female
   ____ Male
   ____ Transgender
   ____ Gender fluid
   ____ Other, please specify _________

2. What is your age? ____ Years

3. What is your race/ethnicity? You may select more than one.
   ____ American Indian/Alaska Native
   ____ Asian
   ____ Black/African American
   ____ Hispanic or Latino
   ____ Native Hawaiian or Other Pacific Islander
   ____ White
   ____ Other, please specify _________

3. What year are you at UNM?
   ____ Freshman
   ____ Sophomore
   ____ Junior
   ____ Senior
   ____ Graduate student
   ____ Other (please specify): ___________________

Over the last **two weeks (14 days)**, how often have you been bothered by the following problems?	Not at all	Several days	More than half the days	Nearly every day
4. Feeling nervous, anxious, or on edge	0	1	2	3
5. Not being able to stop or control worrying	0	1	2	3
6. Worrying too much about different things	0	1	2	3
7. Trouble relaxing	0	1	2	3
8. Being so restless that it's hard to sit still	0	1	2	3
9. Becoming easily annoyed or irritable	0	1	2	3
10. Feeling afraid as if something awful might happen	0	1	2	3

11. If you checked off any problems, how difficult have these problems made it for you to do your work, take care of things at home, or get along with other people?

______ Not difficult at all
______ Somewhat difficult
______ Very difficult
______ Extremely difficult

For each statement, select the response that best matches how much you agree with the statement:

12. I love food
    Strongly disagree Disagree Neither agree nor disagree Agree Strongly Agree
13. I often decide that I don’t like a food, before tasting it
    Strongly disagree Disagree Neither agree nor disagree Agree Strongly Agree
14. I enjoy eating
    Strongly disagree Disagree Neither agree nor disagree Agree Strongly Agree
15. I look forward to mealtimes
    Strongly disagree Disagree Neither agree nor disagree Agree Strongly Agree
16. I eat more when I’m annoyed
    Strongly disagree Disagree Neither agree nor disagree Agree Strongly Agree
17. I often notice my stomach rumbling
    Strongly disagree Disagree Neither agree nor disagree Agree Strongly Agree
18. I refuse new foods at first
    Strongly disagree Disagree Neither agree nor disagree Agree Strongly Agree
19. I eat more when I’m worried
    Strongly disagree Disagree Neither agree nor disagree Agree Strongly Agree
20. If I miss a meal I get irritable
    Strongly disagree Disagree Neither agree nor disagree Agree Strongly Agree
21. I eat more when I am upset
    Strongly disagree Disagree Neither agree nor disagree Agree Strongly Agree
22. I often leave food on my plate at the end of a meal
    Strongly disagree Disagree Neither agree nor disagree Agree Strongly Agree
23. I enjoy tasting new foods
    Strongly disagree Disagree Neither agree nor disagree Agree Strongly Agree
24. I often feel hungry when I am with someone who is eating
    Strongly disagree Disagree Neither agree nor disagree Agree Strongly Agree
25. I often finish my meals quickly
    Strongly disagree Disagree Neither agree nor disagree Agree Strongly Agree
26. I eat less when I’m worried
    Strongly disagree Disagree Neither agree nor disagree Agree Strongly Agree
27. I eat more when I am anxious
    Strongly disagree Disagree Neither agree nor disagree Agree Strongly Agree
28. Given the choice, I would eat most of the time
    Strongly disagree Disagree Neither agree nor disagree Agree Strongly Agree
29. I eat less when I’m angry
    Strongly disagree Disagree Neither agree nor disagree Agree Strongly Agree

30. I am interested in tasting new food I haven't tasted before
    Strongly disagree Disagree Neither agree nor disagree Agree Strongly Agree
31. I eat less when I'm upset
    Strongly disagree Disagree Neither agree nor disagree Agree Strongly Agree
32. I eat more when I'm angry
    Strongly disagree Disagree Neither agree nor disagree Agree Strongly Agree
33. I am always thinking about food
    Strongly disagree Disagree Neither agree nor disagree Agree Strongly Agree
34. I often get full before my meal is finished
    Strongly disagree Disagree Neither agree nor disagree Agree Strongly Agree
35. I enjoy a wide variety of foods
    Strongly disagree Disagree Neither agree nor disagree Agree Strongly Agree
36. I am often last a finishing a meal
    Strongly disagree Disagree Neither agree nor disagree Agree Strongly Agree
37. I eat more and more slowly during the course of a meal
    Strongly disagree Disagree Neither agree nor disagree Agree Strongly Agree
38. I eat less when I'm annoyed
    Strongly disagree Disagree Neither agree nor disagree Agree Strongly Agree
39. I often feel so hungry that I have to eat something right away
    Strongly disagree Disagree Neither agree nor disagree Agree Strongly Agree
40. I eat slowly
    Strongly disagree Disagree Neither agree nor disagree Agree Strongly Agree
41. I can't eat a meal if I have had a snack just before
    Strongly disagree Disagree Neither agree nor disagree Agree Strongly Agree
42. I get full easily
    Strongly disagree Disagree Neither agree nor disagree Agree Strongly Agree
43. I often feel hungry
    Strongly disagree Disagree Neither agree nor disagree Agree Strongly Agree
44. When I see or smell food that I like, it makes me want to eat
    Strongly disagree Disagree Neither agree nor disagree Agree Strongly Agree
45. If my meals are delayed I get light-headed
    Strongly disagree Disagree Neither agree nor disagree Agree Strongly Agree
46. I eat less when I'm anxious
    Strongly disagree Disagree Neither agree nor disagree Agree Strongly Agree
